# Ethnomedicinal Study and Evaluation of the Anxiolytic-like and Diuretic Effects of the Orchid *Stanhopea tigrina* Bateman ex Lindl—(Orchidaceae)

**DOI:** 10.3390/ph17050588

**Published:** 2024-05-06

**Authors:** Rocío del Carmen Díaz-Torres, Eunice Yáñez-Barrientos, José Ángel Montes-Rocha, David Jeremías Morales-Tirado, Clara Alba-Betancourt, Deisy Gasca-Martínez, Maria L. Gonzalez-Rivera, María del Carmen Juárez-Vázquez, Martha Alicia Deveze-Álvarez, Mario Alberto Isiordia-Espinoza, Candy Carranza-Álvarez, Angel Josabad Alonso-Castro

**Affiliations:** 1 Multidisciplinary Graduate Program in Environmental Sciences, Autonomous University of San Luis Potosí, San Luís Potosí 78000, Mexico; rocio.diaz@uaslp.mx; 2 Department of Chemistry, University of Guanajuato, Guanajuato 36200, Mexico; eybarrientos@ugto.mx (E.Y.-B.); dj.moralestirado@ugto.mx (D.J.M.-T.); 3 School of Professional Studies Huasteca Zone, Autonomous University of San Luis Potosí, Ciudad Valles, San Luís Potosí 79059, Mexico; angel.montes@uaslp.mx; 4 Department of Pharmacy, Division of Natural and Exact Sciences, University of Guanajuato, Guanajuato 36200, Mexico; c.albabetancourt@ugto.mx (C.A.-B.); leonor.glez.rivera@outlook.com (M.L.G.-R.); carmenjuarezv@gmail.com (M.d.C.J.-V.); deveze@ugto.mx (M.A.D.-Á.); 5 Institute of Neurobiology, National Autonomous University of Mexico, Juriquilla 76230, Mexico; gasca@inb.unam.mx; 6 Medical Sciences Research Institute, University of Guadalajara, Tepatitlan, Guadalajara 472620, Mexico; mario.isiordia162@yahoo.com

**Keywords:** *Stanhopea tigrina*, antioxidant activity, anxiolytic activity, diuretic effect

## Abstract

*Stanhopea tigrina* Bateman ex Lindl. (Orchidaceae) is an orchid endemic to Mexico, known as “Calavera” or “calaverita”, in the Huasteca Potosina (central region of Mexico). This plant species is used for the folk treatment of mental disorders and urological kidney disorders, according to the ethnomedicinal information obtained in this study. Ethanolic extracts of leaves (HE) and pseudobulb (PE) were obtained by microwave-assisted extraction (MAE). Gas Chromatography coupled with Mass Spectrometry (GC-MS) was used to carry out the chemical characterization of HE and PE. The pharmacological effects (antioxidant, diuretic, anxiolytic, locomotor, hypnotic, and sedative) of HE and PE were evaluated. The possible mechanism of action of the anxiolytic-like activity induced by HE was assessed using inhibitors of the GABAergic, adrenergic, and serotonergic systems. The possible mechanism of the diuretic action of HE was assessed using prostaglandin inhibitory antagonists and nitric oxide synthase (NOS) blockers. HE at 50 and 100 mg/kg exerted anxiolytic-like activity without inducing hypnosis or sedation. Flumazenil, prazosin, and ketanserin inhibited the anxiolytic-like activity shown by HE, which suggests the participation of GABA, α_1_-adrenergic receptors, and 5-HT_2_ receptors, respectively. The diuretic effect was reversed by the non-selective NOS inhibitor L-NAME, which caused the reduction in nitric oxide (NO). These results demonstrate that the ethanolic extract of *S. tigrina* leaves exhibited anxiolytic-like activity and diuretic effects without inducing hypnosis or sedation. This work validates the medicinal uses of this orchid species.

## 1. Introduction

Anxiety is a mental disorder that interferes with daily life, causing a loss in productivity and decreasing quality of life; the number of patients diagnosed with general anxiety has been increasing worldwide in recent years [1]. Available anxiolytics are often ineffective, produce adverse effects that can compromise people’s safety, and have addictive potential [2]. Therefore, searching for new compounds with neuropharmacological activity is essential. An alternative to consider is the use of natural products, including medicinal plants, as sources of bioactive compounds [3]. The Orchidaceae family has around 800 genera, 30,000 wild species, and 100,000 hybrids worldwide [4]. In Mexico, around 1260 orchid species and 170 genera have been recorded [5]. *Vanilla planifolia* is used in traditional medicine, and its active compound vanillin exerts diuretic, antimicrobial, anxiolytic, and antioxidant activities [6], whereas *Geodorum densiflorum* (Lam) Schltr showed sedative and anxiolytic effects [7]. Active compounds have been isolated from medicinal orchids, such as cymbidin A, from the orchid *Cymbidium goeringii*, which showed a diuretic effect [8]. The compound 2,7-dihydroxy-3,4,9-trimethoxyphenantrene, isolated from the roots of *Laelia anceps*, showed vasorelaxant and antihypertensive effects [9].

The use of medicinal orchids is widespread throughout the world; however, there is little information on the number of species and ethnomedicinal uses because traditional knowledge has only been transmitted orally to their descendants [10]. Ethnobotanical studies can provide information on the level of knowledge of the medicinal uses of plants, how they are used, and their availability. Therefore, it is of great importance to document knowledge with ethnopharmacological studies to validate the traditional knowledge of indigenous peoples [11].

There are 13 species of the genus *Stanhopea*, a member of the Orchidaceae family, including *S. hernandezii*, *S. intermedia*, *S. maculosa*, *S. ruckeri*, *S. martiana*, *S. dodsonian*, *S. radiosa*, *S. saccata*, *S. pseudoradiosa*, *S. graveolens*, *S. oculata*, *S. whittenii,* and *S. tigrina* [12]. Of these species, *S. oculata* and *S. hernandezii* are the only species of this genera with medicinal uses, including the reduction in abdominal pain in women and anti-fatigue activity [13]. *Stanhopea tigrina* is an endemic species of Mexico commonly known as “torito morado” or “calavera” in the Huasteca Potosina (central region of Mexico). This plant species has two epiphytic or lithophytic growth habits, reaches a height that varies between 40 and 70 cm, and possesses globose pseudobulbs, a single terminal leaf, and flexuous roots [14]. The sweet and penetrating aroma of its flowers is due to a combination of terpenes and other aromatic compounds, such as phenyl ethyl acetate, (E)-cinnamyl acetate, acetate of benzyl, and methyl salicylate; the strong fragrance of *Stanhopea tigrina* attracts its pollinator, the male bee *Euglossa viridissima* [12]. The distribution of this species covers the states of Tamaulipas, Veracruz, Hidalgo, Oaxaca, Puebla, Querétaro, and San Luis Potosí, mainly along the Sierra Madre Oriental, between 1000 and 1500 m above sea level [15]. This plant species grows in medium evergreen forests, humid oak forests, mesophilic forests of the Sierra Madre Oriental, Cuetzalan, Sierra Norte de Oaxaca-Mixe, and pine and oak forests [15].

In this work, *S. tigrina* was documented for the folk treatment of nervous breakdowns and/or anxiety and kidney-related diseases in the Huasteca Potosina. To validate its medicinal use in mental disorders, the anxiolytic, locomotor, hypnotic, and sedative activity was evaluated, whereas the medicinal use for renal/urological disorders was assessed using a diuretic model in mice.

## 2. Results

### 2.1. Ethnomedicinal Study

The flea markets from the municipalities of Xilitla, Aquismón, and Tamasopo, belonging to the Huasteca Potosina region, were visited. Xilitla is inhabited mainly by the Nahuatl ethnic group, whereas Aquismón and Tamasopo municipalities are inhabited by the Tenek and Pames groups, respectively. These ethnic groups live in communities with a high degree of marginalization, are difficult to access, and have few or no public health services. On weekends, the inhabitants go to the municipal capitals to sell their products, including *S. tigrina*. The orchid is sold for its medicinal use in mental disorders and urological kidney disorders. A total of 22 people participated in this interview; 72% of the informants corresponded to the female gender and 28% to the male gender, with an average age of 37.9 years for women and 49.6 years for men. The main occupations for women and men were commerce and agriculture, respectively. All participants had a basic level of education. Of the total number of people interviewed, 31.81% use *S. tigrina* as a medicinal plant and 68.18% as an ornamental plant. With a significant use level index, TRAMIL = 32%, a value that can be considered significant for cultural acceptance. The medicinal uses or magical–religious purposes mentioned were as follows: tranquilizer/nervousness (57.14%); evil eye (28.57%); and renal/urological diseases (14.29%). Leaves (71.43%), pseudobulbs (14.29%), and complete plants (14.29%) were the plant organs used in the medicinal preparation of *S. tigrina*. The X^2^ test showed significant differences between the *S. tigrina* organs used by the municipality (X^2^ = 23.818; df = 3, and *p* < 0.001). The species is 71.43% obtained by collecting and 28.57% by backyard cultivation. Informants reported that the form of preparation of the orchid was by decoction and orally administered.

According to the ethnomedicinal study, the leaves of *S. tigrina* are used as a tranquilizer for nervous breakdowns and/or anxiety, whereas the pseudobulb of this orchid is used for kidney-related diseases. Therefore, extracts from the two organs of the orchid were made for further pharmacological evaluation.

### 2.2. Chemical Characterization of S. tigrina Extracts

Table 1 and Table 2 show the list of possible compounds found in *S. tigrina* extracts. The deconvoluted spectra and retention indexes obtained for derivatized compounds in the sample were compared with those reported in the libraries.

**Figure 1:**
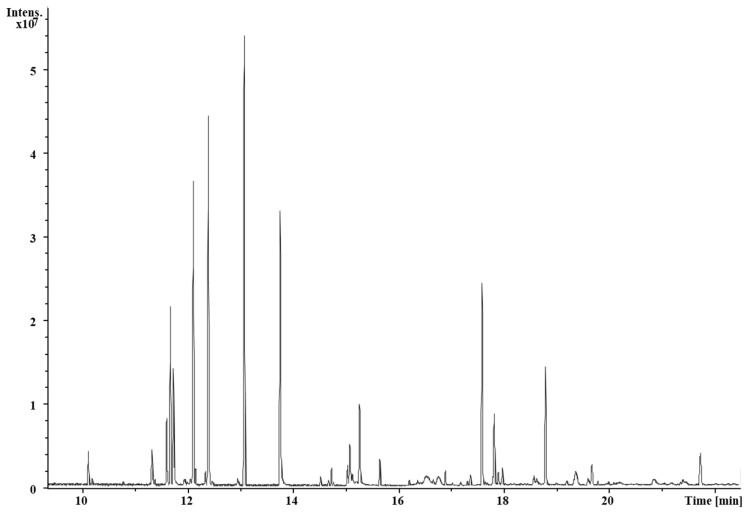
Chromatogram of ethanolic extract of *S. tigrina* leaves (HE) obtained by GC-MS. The detected peaks correspond to the information in Table 1.

Thirty-eight compounds were identified in the ethanol extract of *Stanhopea tigrina* leaves (HE) (Table 1). The signals with the highest intensity were found at retention times of 17.59 and 18.78 min. However, it was not possible to identify these compounds. The compounds **5, 8, 14, 17, 20, 25, 30, 35**, and **38** have been reported with biological activities (Figure 1). Esters, carbohydrates, phenolic acids, and alkaloids were the most abundant compounds in HE (Table 1).

**Figure 2:**
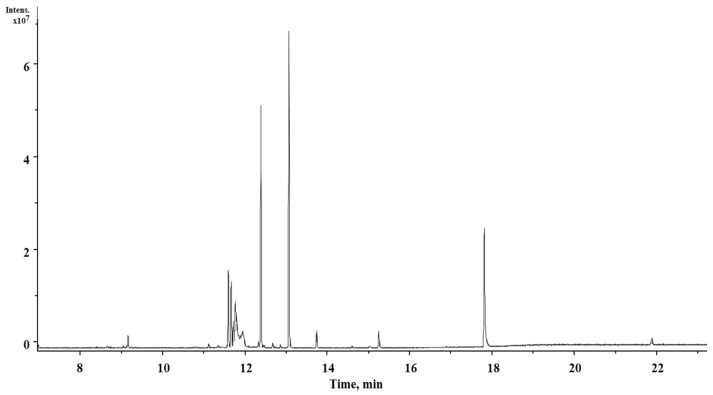
Chromatogram of the ethanolic extract of *S. tigrina* pseudobulbs (PE) obtained by GC-MS. The detected peaks correspond to the information in Table 2.

Twenty-five compounds were identified in the ethanol extract of *Stanhopea tigrina* pseudobulb (PE) (Table 2). The most abundant compounds in PE are carbohydrates and esters. The compounds **6, 13, 14**, and **15** are those for which some biological activities have been reported. In most of these compounds, there are signals with low intensity to the naked eye that are not visible in the chromatogram (Figure 2).

### 2.3. Determination of In Vitro Antioxidant Capacity Using ABTS and DPPH

HE presented the highest antioxidant activity, with an Inhibitory Concentration 50 (IC_50_) value of 3.53 mg/mL for ABTS and an IC_50_ of 0.39 mg/mL in DPPH. PE showed an IC_50_ of 4.57 mg/mL in ABTS and 3.04 mg/mL in DPPH. Ascorbic acid as a positive control presented an IC_50_ of 0.12 mg/mL in ABTS and 0.059 mg/mL in DPPH.

### 2.4. Acute Toxicity Assessment

The acute toxicity revealed that both extracts showed LD_50_ values higher than 2000 mg/kg p.o.; no visible signs of toxicity were appreciated in the mice, and no visible signs of toxicity were observed in liver, lung, heart, intestines, and bladder.

### 2.5. Test to Determine the Anxiolytic and Sedative Activity of Extracts of S. tigrina

#### 2.5.1. Exploratory Cylinder Test

**Figure 3:**
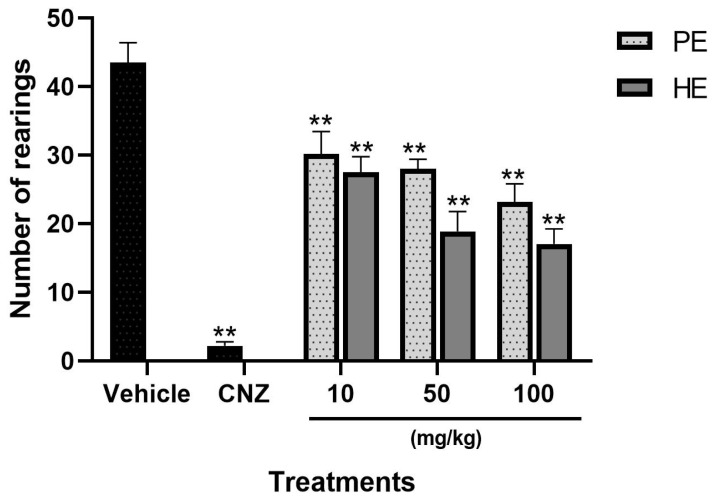
Exploratory cylinder test with *S. tigrina* extracts. Mean ± SEM, ANOVA with Dunnet post hoc test using Statistica software version 13. PE: pseudobulb ethanolic; HE: leaf ethanol extract; CNZ: clonazepam; and Vehicle: saline solution. ** *p* < 0.05 with respect to the vehicle group.

PE and HE showed significant differences compared to the vehicle group (*p* < 0.05). A reduction in the number of rearings was observed, with a dose-dependent anxiolytic-like effect, in HE by 36.79% (10 mg/kg), 54.95% (50 mg/kg), and 60.92% (100 mg/kg) and in PE by 30.7% (10 mg/kg), 35.7% (50 mg/kg), and 46.8% (100 mg/kg). CNZ (1.5 mg/kg) reduced (*p* < 0.05) the number of rearings in the exploratory cylinder, indicating a low state of anxiety-like behavior (Figure 3). The effective dose 50 (ED_50_) for HE was 49.34 mg/kg.

#### 2.5.2. Elevated plus Maze

**Figure 4:**
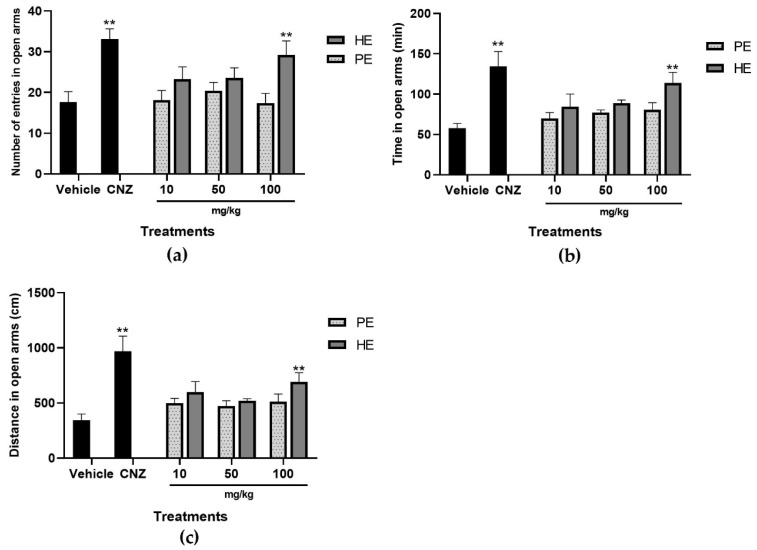
Elevated plus maze (EPM) test with extracts of *S. tigrina*. (**a**) Number of entries in open arms; (**b**) time in open arms; (**c**) distance in open arms. Mean ± SEM, ANOVA with Dunnet’s post hoc test using Statistica software version 13. PE: pseudobulb ethanol extract; HE: leaf ethanol extract; CNZ: Clonazepam; and Vehicle: saline solution. ** *p* < 0.05 compared to the vehicle group.

The results showed that only 100 mg/kg HE increased (*p* < 0.05) the number of entries in open arms (Figure 4a), the time in open arms (Figure 4b), and the distance in open arms (Figure 4c) in the elevated plus maze, and this activity was comparable to that shown by 1.5 mg/kg CNZ, which indicates a lower state of anxiety-like behavior in the mouse.

However, PE at all doses showed no significant effects in the variables of time in open arms (Figure 4). CNZ 1.5 mg/kg increased (*p* < 0.05) the number of entries (Figure 4a), the time (Figure 4b), and the distance (Figure 4c) in open arms.

#### 2.5.3. Light–Dark Box Test

**Figure 5:**
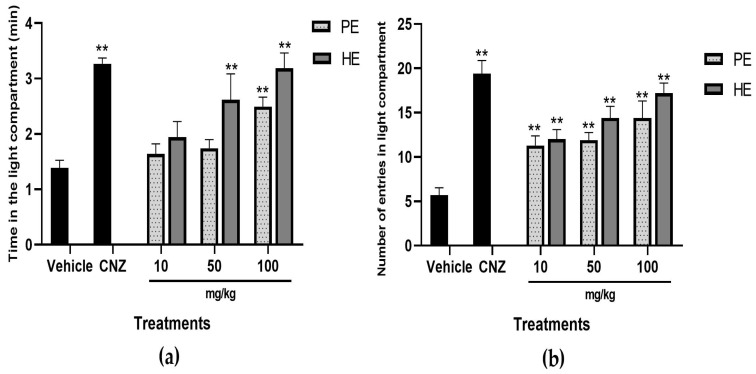
Light–dark box test with extracts of *S. tigrina*. (**a**) Time in the light compartment; (**b**) number of entries in the light compartment. Mean ± SEM, ANOVA with Dunnet’s post hoc test using Statistica software version 13. PE: pseudobulb ethanol extract; HE: leaf ethanol extract; CNZ: clonazepam; and Vehicle: saline solution. ** *p* < 0.05 compared to the vehicle group.

In the light–dark test, HE at 50 and 100 mg/kg and 100 mg/kg PE increased (*p* < 0.05) the time in the light compartment; such effects were comparable to those observed by 1.5 mg/kg CNZ (Figure 5a). All doses of PE and HE showed significant differences (*p* < 0.05) in the number of entries into the light compartment (Figure 5b).

#### 2.5.4. Open-Field Test

HE (100 mg/kg) increased (*p* < 0.05) the time and distance in central squares, whereas PE (100 mg/kg) increased (*p* < 0.05) the distance in central squares (Table 3). These findings suggest an anxiolytic-like behavior. However, HE and PE did not affect locomotor activity (Table 3). CNZ (1.5 mg/kg) decreased (*p* < 0.05) the total distance and increased (*p* < 0.05) the resting time (Table 3), which indicates an imbalance in locomotor activities and sedative effects. CNZ (1.5 mg/kg) increased (*p* < 0.05) the time and distance in central squares (Table 3).

#### 2.5.5. Hole Board Test

**Figure 6:**
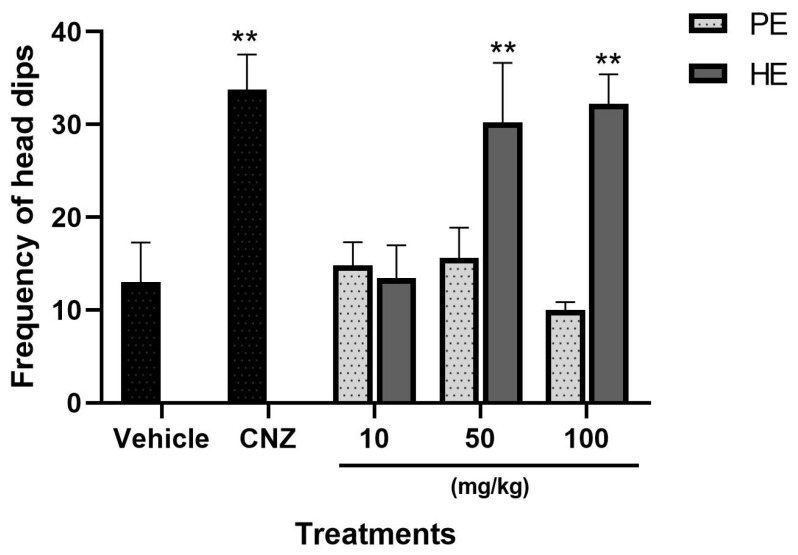
Hole Board Test with extracts of *S. tigrina*. Mean ± SEM, ANOVA with Dunnet’s post hoc test using Statistica software version 13. PE: pseudobulb ethanolic extract; HE: leaf ethanol extract; CNZ: clonazepam; and Vehicle: saline solution. ** *p* < 0.05 compared to the vehicle group.

The number of head dippings recorded in 50 mg/kg and 100 mg/kg HE presented significant differences compared to the vehicle group (*p* < 0.05). CNZ increased the number of head dippings on the board, indicating a lower state of anxiety-like behavior. The anxiolytic-like effect shown by HE was 40% (10 mg/kg), 89% (50 mg/kg), and 96% (100 mg/kg), with an ED_50_ of 11.52 mg/kg (Figure 6). HE at 50 mg/kg and 100 mg/kg presented similar anxiolytic-like activity to that shown by 1.5 mg/kg CNZ. PE at doses of 10, 50, and 100 mg/kg, and 10 mg/kg HE showed no significant anxiolytic-like effects (Figure 6).

#### 2.5.6. Mechanism of Action for Anxiolytic-like Behavior

**Figure 7:**
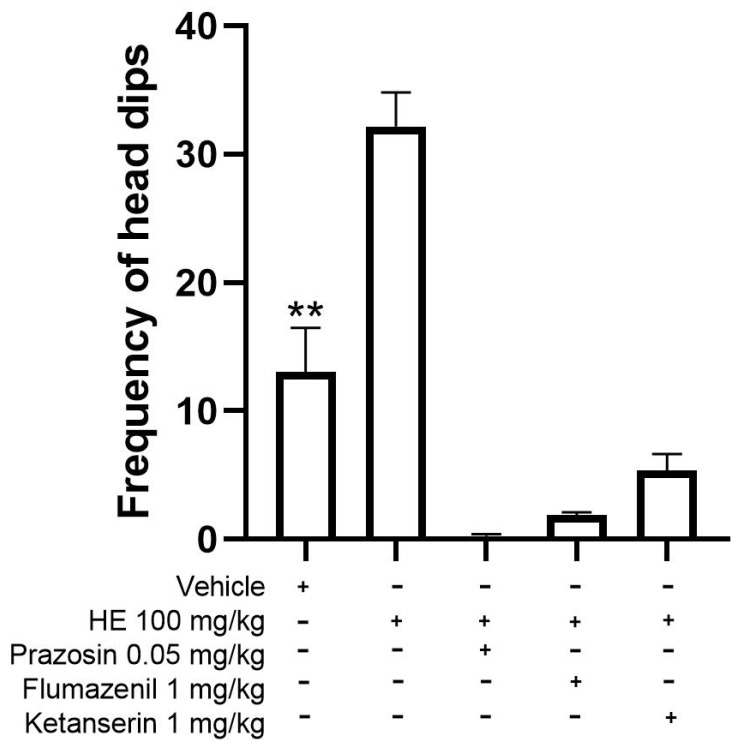
Possible mechanism of action of the HE in the hole board test. Mean ± SEM, ANOVA with Dunnet’s post hoc test using Statistica software version 13. **p < 0.05 compared to the vehicle group.

The possible mechanism of action of the anxiolytic-type activity was carried out with the hole board test with 100 mg/kg HE because this dose showed similar activity to 1.5 mg/kg CNZ. Pretreatments with 0.5 mg/kg prazosin, 1 mg/kg flumazenil, and 1 mg/kg ketanserin inhibited (*p* < 0.05) the anxiolytic-like activity presented by HE (Figure 7).

#### 2.5.7. Rotarod Test

**Figure 8:**
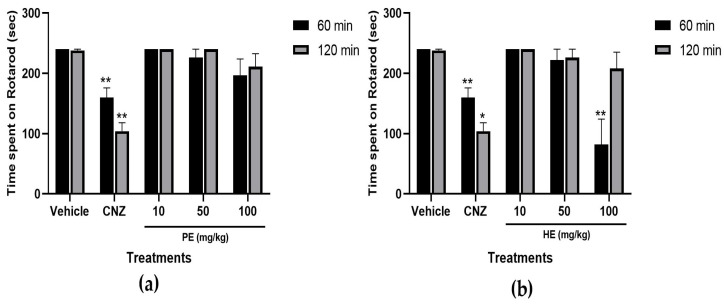
Rotarod test with *S. tigrina* extracts at 60 and 120 min. (**a**) Time spent on Rotarod of PE; (**b**) time spent on Rotarod of HE. Mean ± SEM, ANOVA with Dunnet’s post hoc test using Statistica software version 13. PE: pseudobulb ethanol extract; HE: leaf ethanol extract; CNZ: clonazepam; and Vehicle: saline solution. * *p* < 0.0001 compared to the vehicle group. ** *p* < 0.05 compared to the vehicle group.

The findings in the rotarod test indicated that only 100 mg/kg HE at 60 min post-treatment affected (*p* < 0.05) the locomotor activity of mice (Figure 8b). CNZ reduces the time spent on the rotarod, indicating deterioration in locomotor activity. This effect did not last at 120 min post-treatment. In the rest of the treatments, no significant differences were observed at 60 and 120 min post-treatment (Figure 8a,b).

#### 2.5.8. Hole Cross Test

**Figure 9:**
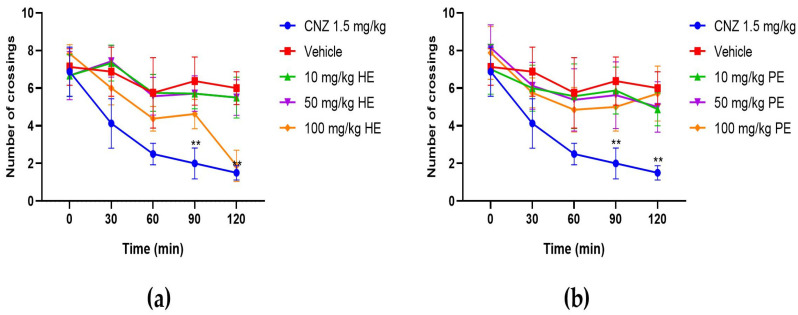
Hole cross test with *S. tigrina* extracts. (**a**) HE extract; (**b**) PE extract. Mean ± SEM, ANOVA with Dunnet post hoc test using Statistica software version 13. PE: pseudobulb ethanolic; HE: leaf ethanol extract; CNZ: clonazepam; and Vehicle: saline solution. ** *p* < 0.05 with respect to the vehicle group.

HE (100 mg/kg) decreased (*p* < 0.05) the number of crossings through the hole at 120 min post-treatment (Figure 9). PE (10–100 mg/kg) did not affect spontaneous locomotion in mice. On the contrary, CNZ (1.5 mg/kg) decreased the locomotion activity of mice from 60 min to 120 min post-treatment (Figure 9).

#### 2.5.9. Pentobarbital-Induced Sleep Test

**Figure 10:**
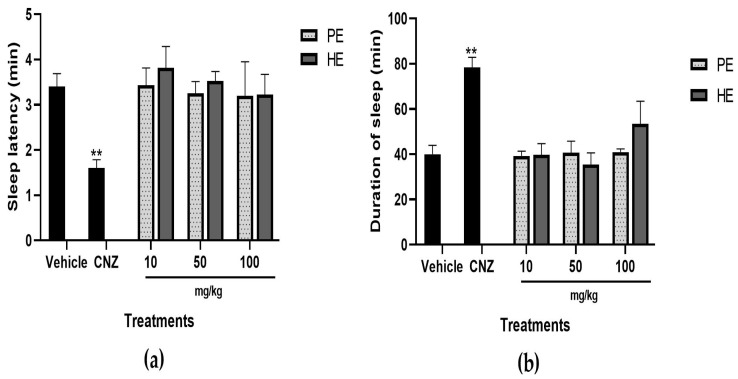
Pentobarbital-induced sleep test with extracts of *S. tigrina*. Sleep latency (**a**) and duration of sleep (**b**). Mean ± SEM, ANOVA with Dunnet’s post hoc test using Statistica software version 13. PE: pseudobulb ethanol extract; HE: leaf ethanol extract; CNZ: clonazepam; and Vehicle: saline solution. ** *p* < 0.05 compared to the vehicle group.

PE and HE did not show significant differences compared to the vehicle in the sleep latency (Figure 10a) and the duration of sleep (Figure 10b), which indicates that PE and HE showed no sedative or hypnotic activity (Figure 10). A slight but non-significant increase in sleep duration was observed for 100 mg/kg HE (Figure 10b). CNZ decreased (*p* < 0.05) sleep latency time and increased (*p* < 0.05) sleep duration; this indicates sedative and hypnotic activities.

#### 2.5.10. Anticonvulsant Activity

Convulsion duration decreased (*p* < 0.05), and the onset of convulsions increased (*p* < 0.05) with 100 mg/kg HE (Table 4). In the percentage of mortality, no significant differences were observed (*p* > 0.05), and the PE and HE treatments did not show a similar effect to CNZ (Table 4). CNZ (1.5 mg/kg) protected all animals from convulsions (Table 4).

### 2.6. Diuretic Effect

**Figure 11:**
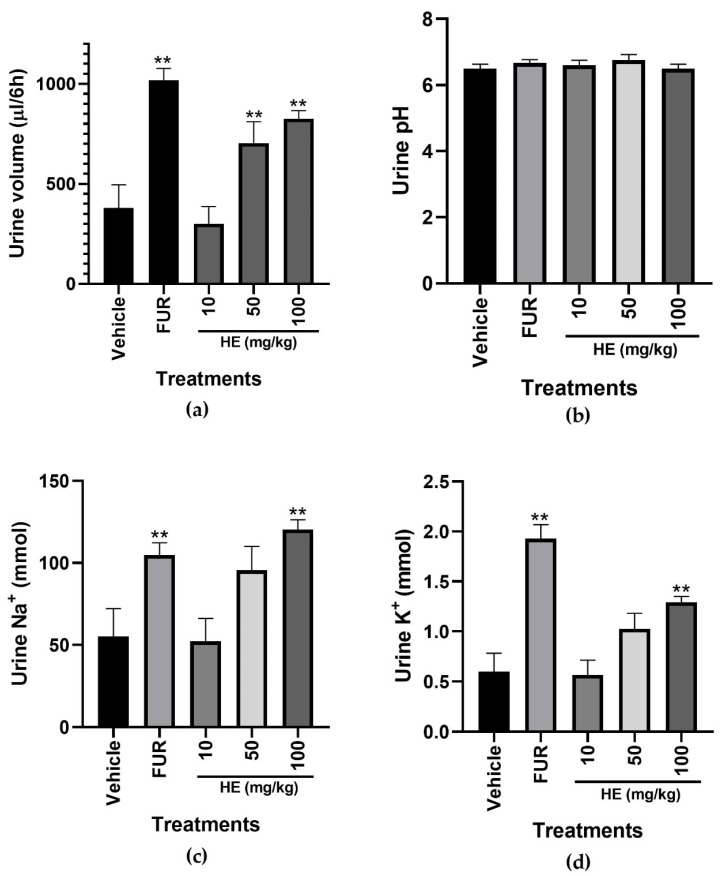
Diuretic Effect Test to the HE extract of *S. tigrina*. (**a**) Urine volume; (**b**) urine pH; (**c**) urine Na^+^; and (**d**) urine K^+^. Mean ± SEM, ANOVA with Dunnet’s post hoc test using Statistica software version 13. Urine pH. Mean ± SEM, ANOVA with Tukey’s multiple comparisons post hoc test. PE: pseudobulb ethanolic; HE: leaf ethanol; FUR: furosemide; and Vehicle: saline solution. ** *p* < 0.05 compared to the vehicle group.

The diuretic effect of PE and HE was evaluated. PE did not increase urine excretion; therefore, measurement of pH and electrolytes Na^+^ and K^+^ were not performed (results not shown). HE at 50 and 100 mg/kg increased (*p* < 0.05) the volume of urine excreted, with a significant difference compared to the vehicle group (Figure 11a). The pH values of urine collected with the HE treatment did not show significant differences between treatments (*p* < 0.05) (Figure 11b). HE (100 mg/kg) increased (*p* < 0.05) the excretion of Na^+^ and K^+^; the excretion of Na^+^ with 100 mg/kg HE showed a similar activity compared to that shown by 10 mg/kg FUR (Figure 11c), and the excretion of K^+^ with 100 mg/kg showed lower activity compared to the positive control (Figure 11d).

#### Mechanism of Action for the Diuretic Effect

**Figure 12:**
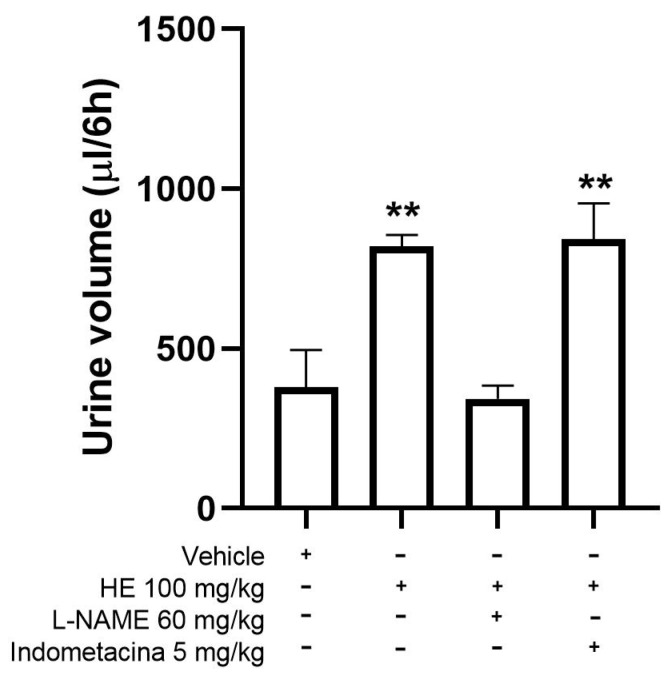
Possible mechanism of action of the diuretic effect shown by HE. Mean ± SEM, ANOVA with Dunnet’s post hoc test using Statistica software version 13. HE: leaf ethanol extract. ** *p* < 0.05 compared to the vehicle group.

The possible mechanism of action of the diuretic activity of HE was evaluated with two antagonist drugs: L-NAME at 60 mg/kg; and Indomethacin at 5 mg/kg. Pretreatment with Indomethacin did not show significant differences with HE, whereas L-NAME inhibited (*p* < 0.05) the diuretic effect shown by HE (Figure 12).

## 3. Discussion

The medicinal uses of *S. tigrina* obtained using the ethnomedicinal study revealed its pharmacological potential. This species is used in the Huasteca Potosina as a tranquilizer for mental disorders, mainly in anxiety crises and, to a lesser extent, in diseases associated with the kidneys. The UST index = 32% reveals that the use given to *S. tigrina* has cultural acceptance. According to Jiménez-Romero et al. [16], a level of significant use tramil (UST) greater than or equal to 20% is a significant value of cultural acceptance, and the species can be considered for scientific validation. Other orchids are used in traditional medicine for the folk treatment of anxiety and related diseases to the kidney. For instance, *Geodorum densiflorum* and *Coelogyne suaveolens* showed anxiolytic and sedative effects in preclinical studies [7,8], and *Vanilla planifolia* demonstrated anxiolytic-like activity and diuretic effect [6].

This is the first study reporting the chemical composition obtained by GC-MS of the ethanolic extracts of leaf (HE) and pseudobulb (PE) of *S. tigrina*. The chromatogram of HE showed the presence of (**8**)Neophytadiene, a diterpene that presented anxiolytic, anticonvulsant, and antidepressant effects, without sedative or locomotor effects, with probable participation of the GABAergic system [11]. Another compound present in HE is (**35**)5,12-Dimethoxy-2,3,8,9-tetramethoxybenzo[c]phenanthridin-6(5H)-one, an isoquinoline alkaloid reported with an inhibitory effect on β-amyloid aggregation, which is involved in neurodegenerative Alzheimer’s disease [17]. (**25**)Cinnamic acid, p-(trimethylsiloxy)-, trimethylsilyl ester, presents neuroprotective properties [18].

Some of the compounds found in PE include ^(**13**)^1-phenylpyrrolo [2,1,5-cd]indolizine and ^(**14**)^1-(p-Acetylbenzoyl)pyrrolidine, containing the pharmacophore structure pyrrolo [2,1,5-cd]indolizine reported with neuroprotective activity [19]. ^(**15**)^Caffeine, an alkaloid associated with diuretic activity [20], decreases the rate of kidney stones and activates the central nervous system (SNC) [21].

HE and PE exerted low (LD_50_ > 2000 mg/kg p.o.) acute toxicity in mice without apparent signs of toxicity (loss in weight, piloerection, immobility, salivation, etc.) after 14 days of a single-dose treatment. The orchid *Cyrtopodium macrobulbon* showed antinociceptive activity and low acute toxicity (LD_50_ > 5000 mg/kg p.o.) [22], whereas the orchid *Nervilia purpurea* showed analgesic and anti-inflammatory effects with LD_50_ > 15 g/kg (p.o.) [23]. Those studies indicate that orchids have low toxicity in acute assays.

The production of reactive oxygen species (ROS) causes oxidative stress and damages neuronal membranes, leading to mental disorders like anxiety [24]. Some clinical and preclinical studies have reported that oxidative stress at the peripheral level is associated with anxiety symptoms [24]. In the evaluations of antioxidant capacity, HE showed greater ABTS activity (IC_50_ = 3.53 mg/mL) and DPPH (IC_50_ = 0.39 mg/mL) activity compared to the PE extract but lower compared to the positive control ascorbic acid ABTS (IC_50_ = 0.12 mg/mL) and DPPH (IC_50_ = 0.059 mg/mL).

The anxiolytic-like activity was evaluated with the following tests: exploratory cylinder; hole board; elevated plus maze; light–dark box; and open field. These tests are accepted and used to study behavior characterized by exploration (wandering and rearing), considering that fear and curiosity related to anxious behavior are expressed by animals in an unknown environment [25]. CNZ, belonging to the benzodiazepine family, was used as a positive control due to its inhibitory effects on gamma-aminobutyric acid (GABA). However, CNZ induces sedative and hypnotic effects and exerts impairment in locomotor activity [26].

In the hole board test and the exploratory cylinder test, anxiety-like behavior was evaluated through exploratory behavior, and reductions in exploratory rearings and head dippings in the exploratory cylinder test and hole board test, respectively, indicate anxiolytic-like effects [25]. HE and PE (10–100 mg/kg p.o.) each showed dose-dependent anxiolytic-like effects in the hole board test and the exploratory cylinder test. The anxiolytic-like activity of 50 and 100 mg/kg HE in the hole board test was comparable to that shown by 1.5 mg/kg CNZ. PE showed no anxiolytic-like activity on this test.

The elevated plus maze test assesses anxious behavior in mice; based on their natural aversion to exploring the open arms of the maze, this test is useful for the detection of anxiolytic-like drugs that increase the number of entries, distance, and time in the open arms [27]. HE at 100 mg/kg decreased anxiety-like behavior by increasing the number of entries and the distance traveled in the open arms, an effect equivalent to 1.5 mg/kg CNZ. PE did not induce an anxiolytic-like effect.

The light–dark test evaluates the anxiolytic-like effect based on the mouse’s anxiety-like behavior in illuminated areas; the presence of anxiolytic-like compounds causes an increase in the time spent in the light compartment [28]. PE (100 mg/kg) and HE (50 and 100 mg/kg) showed anxiolytic-like properties, as evidenced by the increase in time of the light compartment with similar activity to 1.5 mg/kg CNZ. HE and PE increased in a dose-dependent manner the number of entries to the light compartment, with comparable activity to that shown by CNZ (1.5 mg/kg).

The open field test evaluates locomotor activity and anxiety-like behavior based on the conditioned fear of rodents of exploring new environments and facing threatening situations. The tendency of mice to stay close to the walls (thigmotaxis) is an indicator of anxiety-like behavior, whereas greater exploration in the central area is a lower state of anxiety-like behavior in rodents [25]. PE (100 mg/kg) increased the distance toward the central squares, and HE (100 mg/kg) increased the distance traveled and time spent in the central squares. The total distance and resting time were not affected by the treatment with HE or PE, indicating that both plant extracts showed no alterations in locomotor activity in mice like those shown by anxiolytic agents like benzodiazepines [26].

The use of inhibitors of GABAergic, adrenergic, and serotonergic systems involved in the neurotransmission process was used as a strategy to evaluate the possible mechanisms of action of the anxiolytic-like activity of HE. The anxiolytic-like of HE was inhibited by the antagonist drugs flumazenil, prazosin, and ketanserin, which block GABA [28], α_1_-adrenergic receptors (α1-AR) [29], and 5-HT_2_ receptors [30], respectively. The main inhibitory neurotransmitter in the central nervous system (CNS) is gamma-aminobutyric acid (GABA); reducing neuronal activity and regulating cognitive function, emotional behavior, and the body’s response to stress, α1-AR participates in the regulation and function of the central nervous system (CNS), peripheral, and in physiological responses mediated by epinephrine and norepinephrine in the cardiovascular system [31]. Serotonin 5-HT_2_ receptors located in the hippocampus and amygdala participate in the modulation of cognitive functions, human emotion, and regulating anxiety behavior [32]. The antagonist drugs flumazenil, prazosin, and ketanserin inhibited the anxiolytic-like effect of HE, which suggests the participation of GABAergic, adrenergic, and serotonergic systems. The presence of compounds **8** (Neophytadiene), **25** (cinnamic acid), and **35** (5,12-Dimethoxy-2,3,8,9-tetramethoxybenzo[c]phenanthridin-6(5H)-one) could participate and contribute to the anxiolytic-like effects of HE.

The effects of HE and PE on locomotor activity were evaluated in the rotarod and hole cross tests. The rotarod test evaluates motor coordination and muscle relaxation effects [33], whereas the hole cross test spontaneous locomotor activity [34]. Only in the rotarod test, HE at 100 mg/kg showed a significant decrease, compared to the vehicle group, in locomotor activity reflected by a decrease in time spent on the rotarod at 60 min post-treatment, but this effect was lost at 120 min post-treatment. On the contrary, this effect in locomotor activity was not significant in the hole cross test. CNZ (1.5 mg/kg) decreased locomotion in mice, indicative of sedation and loss in CNS excitability.

The hypnotic and sedative effect of HE and PE was assessed using the pentobarbital-induced sleep test. Pentobarbital induces a hypnotic and sedative effect due to its binding to GABA subtype A receptors [26]. The results suggest that PE and HE did not enhance the hypnotic and sedative effect of pentobarbital. This suggests that both extracts lack sedative and hypnotic actions.

Pentylenetetrazole (PTZ), a GABA antagonist, decreases GABA levels, causing stimulation of cortical neurons and convulsions. CNZ (1.5 mg/kg) inhibited convulsions, and 100 mg/kg HE decreased the seizure onset and the duration of convulsions.

The diuretic effect of *S. tigrina* extracts was evaluated to validate its medicinal use in kidney-associated diseases. Diuretics are used as a treatment for chronic kidney failure, nephrotic syndrome, hypertension, congestive heart failure, pulmonary edema, and cirrhosis [35]. Diuretics are stimulants of the excretion of urine, electrolytes, and toxic agents and can also increase the amount of antibiotics excreted in the urine, promoting renal protective effects [35]. PE lacked diuretic activity (results not shown), and 100 mg/kg HE induced a diuretic effect, with similar activity to that exerted by 10 mg/kg FUR. Loop diuretics block the renal Na/K/2Cl transporter in the loop of Henle, stimulating an increase in urine elimination, a decrease in the volume of fluid in the tissues, and an increase in the excretion of electrolytes [35]. The increase in urine volume is due to the inhibition of the reabsorption of sodium and chloride ions in the renal tubule, generating changes in the renal handling of potassium, calcium, magnesium, and urea [35]. An essential quality of a good diuretic is when the treatment increases sodium excretion to a greater extent than potassium, a characteristic considered a good safety profile due to the reduction in hypokalemia, an adverse side effect of furosemide [35]. HE increased the volume of urine excreted and the urinary elimination of sodium, with a similar effect to that shown by 10 mg/kg FUR. HE also increased the potassium excretion, although with lower activity, compared to 10 mg/kg FUR. The results indicated that HE might act as a loop diuretic, increasing natriuresis and kaliuresis, with a low risk of hypokalemia. The chromatogram of the chemical characterization of HE showed the presence of (**27**)Gallic acid- tetrakis(trimethylsilyl) derivative, a phenolic compound reported as a potassium-sparing diuretic, antihypertensive, and vasorelaxant agent [36]. The presence of this compound may contribute to the diuretic effect observed in HE.

L-nitro arginine methyl ester (L-NAME) and indomethacin, antagonists of nitric oxide and prostaglandins, respectively, were used to determine the possible mechanism of action of the diuretic activity of HE. The diuretic effect of HE was inhibited by the administration of L-NAME, a non-selective inhibitor of nitric oxide synthase (NOS) [37]. The diuretic effect can be attributed to the release of nitric oxide (NO), a mediator of elevated blood pressure at the preglomerular level and increased spinal blood flow, giving rise to diuresis [38]. Intrarenal NO reduces renal vascular resistance (RVR), which is responsible for the regulation of sodium reabsorption by the nephron [38]. Therefore, it is likely that the mechanism of action of the diuretic effect of the HE is associated with the synthesis of NO.

This work validated the therapeutic potential of *S. tigrina* leaves as an anxiolytic-like and diuretic agent used in the Huasteca Potosina (central region of Mexico). This work reported the chemical characterization and the pharmacological effects (anxiolytic-like and diuretic) of ethanol extracts with pseudobulbs and leaves of *S. tigrina*. This study provides information on the medicinal properties of an orchid used in Mexican traditional medicine. Some of the limitations and prospects of this study include the following aspects: (1) The ethnomedicinal study was performed in one region of Mexico; it is necessary to perform further ethnomedicinal studies of *S. tigrina* in other regions of Mexico; (2) The chemical characterization of the plant extracts showed the presence of identified compounds. Further phytochemical work is required to isolate and elucidate the structure of the unknown compounds; (3) Current work is performed in our research group to evaluate the anxiolytic-like and diuretic effects of some compounds identified in the extracts of *S. trigrina* leaves.

## 4. Materials and Methods

### 4.1. Ethnomedicinal Study

The ethnomedicinal study was carried out from January to May 2022 to record the medicinal uses of *S. tigrina* in the Huasteca Potosina region, inhabited by Nahuatl, Tenek, and Pame ethnic groups, who have developed ways of treating diseases with the plant resources obtained from the environment according to their beliefs, customs, and ideologies. The flea markets of Xilitla, Aquismón, and Tamasopo, municipalities of San Luis Potosí, Mexico, located in the Eastern Sierra Madre, were visited. Semi-structured interviews were applied to obtain information regarding *S. tigrina*, such as common name, medicinal use, part used, method of obtaining, preparation, side effects or discomforts, and possible combinations with allopathic medicine. Sociocultural data were also collected, such as gender, age, and occupation. The data were organized into frequencies and percentages of citations of traditional knowledge. The level of significant use was estimated following the TRAMIL methodology [16].

### 4.2. Plant Material

**Figure 13:**
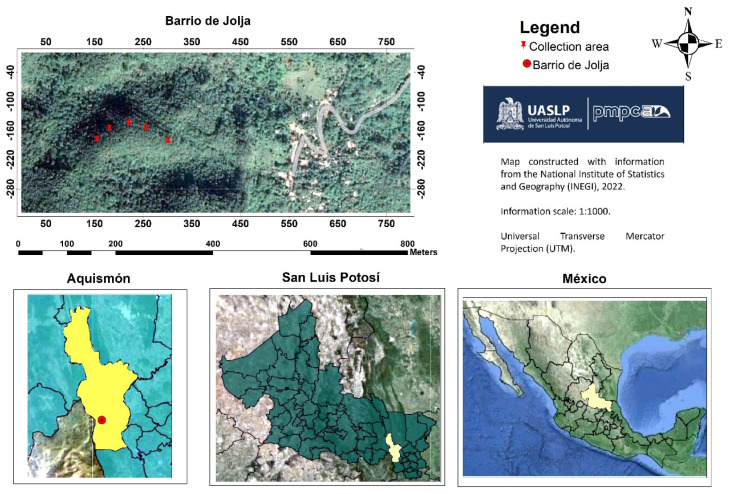
Location of Barrio de Jolja, Tampaxal, Aquismón, SLP, Mexico.

The sample collection of *S. tigrina* was carried out in the locality of Barrio de Jolja, Tampaxal, belonging to the municipality of Aquismón in the state of San Luis Potosi, in July 2021 (Figure 13). The interviews recorded the common name, date, growth habit, geographical coordinates, and information on the medicinal uses attributed to *S. tigrina*. One of the collected specimens was deposited, with voucher number 62290, in the Isidro Palacios Herbarium of the Desert Zones Research Institute (IIZD) of the Autonomous University of San Luis Potosí (UASLP). The plant specimen was identified by Dr. Eleazar Carranza González.

### 4.3. Reagents

Clonazepam (CNZ), furosemide (FUR), flumazenil, prazosin, ketanserin, L-NAME, indomethacin, pyridine, N,O-bis(trimethylsilyl)trifluoroacetamide (BSTFA), 2,2′-azino-bis-3-ethylbenzothiazoline-6-sulfonic acid-free (ABTS), 1,1-diphenyl-2-picrylhydrazyl (DPPH), and ascorbic acid were obtained from Sigma-Aldrich (St Louis, MO, USA).

### 4.4. Plant Extracts

The plant material was dried in a convection oven (Lindberg/Blue M brand, model MO1450A-1, Asheville, NC, USA), at 40 °C for 24 h. The dried plant material was pulverized in an analytical mill (IKA brand model M20, Mason, Ohio, USA). Ethanolic extraction of pseudobulb (PE) and leaves (HE) was carried out using microwave-assisted extraction (Anton Paar brand, Multiwave PRO Microwave Reaction System model, Graz, Austria). A rotavapor system (Büchi brand, model Interface 1–100, Flawil, Switzerland) was used for 2 h to concentrate each extract until dryness. The extracts were resuspended in saline solution (sodium = 154 mmol/L, chlorine =154 mmol/L) for further pharmacological evaluations.

### 4.5. Chemical Characterization

#### 4.5.1. Sample Derivatization

For derivatization, 10 mg of each dried ethanolic extract (PE or HE) was dissolved in 370 µL of pyridine and 50 µL of BSTFA and incubated at 50 °C for 1 h.

#### 4.5.2. GC-MS Analysis

A PerkinElmer Clarus 580 Gas Chromatograph equipped with a column Elite-5 MS (30 m × 0.32 mm, i.d. and 0.25 μm film thickness of coated material) was used for the chemical characterization of plant extracts. All analyses were performed in Full Scan mode. The helium flow rate was 1 mL/min; the injection volume was 1 µL; the injection port was set to split 1:5 and maintained at 250 °C. The column oven temperature was 80 °C for 2 min, then ramped to 300 °C at 12 °C/min and held for 6 min.

Temperature settings for the transfer line heater and ion source of the mass spectrometer were 290 °C and 250 °C, respectively. Spectra were acquired in the *m*/*z* range of 50–620. After a solvent delay of 7 min, the filament was turned on. The column effluent was into the ion source of a PerkinElmer Clarus SQ 8 S Mass Spectrometer (Waltham, MA, USA).

#### 4.5.3. Analysis of GC-MS Data

The analysis of data was performed using the AMDIS software version 2.62 (Automated Mass Spectral Deconvolution and Identification System; http://www.amdis.net/ accessed on 20 April 2024) in “Use Retention Index Data” mode. The identification of compounds was made through MS Search 2.0 software using NIST/EPA/NIH (NIST 2020) and Wiley 12th edition Mass Spectral Libraries.

### 4.6. Determination of the In Vitro Antioxidant Capacity Using ABTS and DPPH

The in vitro antioxidant capacity of each plant extract (HE or PE) tested at 1–7 mg/mL was determined using the 2,2′-azino-bis-3-ethylbenzothiazoline-6-sulfonic acid-free radical scavenging method (ABTS) and the 1,1-diphenyl-2-picrylhydrazyl (DPPH) elimination assay [39], with slight modifications. Briefly, the ABTS assay was performed in a Thermo Scientific UV-visible spectrophotometer and 10 mm thick Hellma acrylic cuvette. A 7 mM ABTS solution was placed in the cuvette and adjusted to an absorbance of 0.70 ± 0.02 at a wavelength of 734 nm. The antioxidant activity was determined using the DPPH radical at an absorbance of 1.8 with a wavelength of 520 nm. The scanning capacity of the extract was compared with that of the standard solution and was calculated from the following formula: ABTS or DPPH-removing activity (%) = (Abs control-Abs Sample)/(Abs control) × 100. Abs control is the absorbance of the blank, and sample Abs is the absorbance in the presence of the samples and the standard solution. The standard curve was performed with an ascorbic acid (AA) solution (0–100 mg/mL). The results were expressed as mg of ascorbic acid/100 g of plant extract. The effective concentration 50 (EC_50_) value was also obtained, which was the concentration of the sample required to eliminate ABTS or DPPH radicalization by 50%.

### 4.7. Experimental Animals

The experiments were carried out following the indications by the Mexican legislation [40]. Male Balb/c mice, six weeks old, weighing 25 to 30 g, were obtained from the bioterium of the Division of Natural and Exact Sciences of the University of Guanajuato at Noria Alta campus. Mice were maintained at 23 ± 2 °C, 55 ± 5% humidity, with 12 h/12 h light and dark cycles. The protocol of this study was revised and approved by the ethical committee for the research of the University of Guanajuato (CIBIUG-P48-2022).

### 4.8. Acute Toxicity Assessment

The acute toxicity of the HE and PE was estimated according to the OECD protocol [41], for which the mice were observed daily for 5 days to record mortality, changes in behavior, and other toxic signs.

### 4.9. Experimental Model

Each experimental group consisted of eight animals per group. All treatments were administered orally. The vehicle group received 100 µL of saline solution (sodium = 154 mmol/L, chloride = 154 mmol/L); clonazepam (CNZ) at 1.5 mg/kg was the positive control group in the tests of anxiolytic, locomotor, hypnotic, and sedative activity, whereas furosemide (FUR) at 10 mg/kg was the positive control for diuretic and the experimental groups, which received PE or HE at 10, 50, and 100 mg/kg. After 60 min of each treatment administration, each behavioral test was performed.

### 4.10. Exploratory Cylinder Test

Each mouse was individually placed in an acrylic cylinder (45 cm high, 20 cm diameter, with a 3 mm wall) with filter paper and constant lighting. The number of lifts performed by each mouse was recorded for 5 min [11].

### 4.11. Hole Board Test

Each mouse was individually placed in the central section of a hole board (50 cm by 50 cm wide and 20 cm high, with 16 holes of two cm diameter). Locomotor activity was evaluated by recording the number of times the mouse inserted its head into the holes up to at least the level of the ears for 5 min. The increase in the number of explorations suggests a lower state of anxiety and greater exploratory activity [25].

### 4.12. Elevated plus Maze

The elevated plus maze (EPM) test evaluates the anxiety-like behavior that each mouse experiences when faced with new environments. It has two closed arms (30 × 5 × 15 cm) with sides and bottom walls that are open at their ends and two open arms measuring 30 × 5 cm, without side or bottom walls. Each mouse was individually placed in the center of the EPM in front of an open arm for free exploration for 5 min. A greater number of entries, time, and distance in the open arms indicates a lower state of anxiety in the mouse [27].

### 4.13. Light–Dark Box Test

The light–dark box (46 × 27 × 30 cm) is divided into three parts; one-third of the box corresponds to the dark compartment and two-thirds to the light compartment with a light intensity of 400 lx. The mice were introduced individually into the light compartment, and the number of entries into the light compartment and the time spent in the light compartment were recorded for 5 min for each mouse. This test allows for the evaluation of anxiety-like behavior [28].

### 4.14. Open-Field Test

Mice were placed individually in the center of the cage (42 × 42 × 30 cm), divided into 25 equal squares, and allowed to explore freely for 5 min; total distance, rest time, time spent in the center, and the distance traveled to the central squares were recorded. The distance traveled evaluates the effect on locomotor activity, whereas the tendency to remain in the periphery, avoiding the central section, is considered an indicator of anxiety-like behavior [25].

### 4.15. Possible Mechanism of Anxiolytic Action

The mechanism of action was evaluated by the intraperitoneal administration of 1 mg/kg flumazenil (GABA antagonist), 0.5 mg/kg prazosin (α1 adrenergic receptor blocker), or 1 mg/kg ketanserin (5-HT_2_ receptor blocker). After 15 min, 100 mg/kg p.o. HE was administered, and 45 min later, the hole board test was performed.

### 4.16. Rotarod Test

Mice capable of walking on the rotating roller at 4 rpm for 4 min were selected, which maintained balance, coordination, and motor planning. The time that each animal remained on the rotating rod was recorded at 60 and 120 min after treatment [33].

### 4.17. Hole Cross Test

A wooden cage (20 × 14 × 30 cm) with a hole of 6 cm in diameter and 6 cm in height from the ground, located in the center of the cage, was used. The number of crossings performed by each animal through the hole from one compartment to the other compartment during 3 min was registered at 30, 60, 90, and 120 min [34].

### 4.18. Pentobarbital-Induced Sleep Test

Each mouse received an intraperitoneal injection of 40 mg/kg of pentobarbital and was placed individually in acrylic cages for observation. Sleep onset was taken as the interval from the intraperitoneal injection of pentobarbital to loss in the righting reflex (when the mouse was able to turn onto its back), whereas sleep duration was the interval from loss in the righting reflex until the recovery of righting reflex [42].

### 4.19. Anticonvulsant Activity

The mice were administered intraperitoneally with 90 mg/kg of pentylenetetrazole (PTZ), dissolved in saline solution, placed individually in acrylic cylinders, and observed for 30 min. The latencies for the onset and duration of the clonic seizure and the mortality of the mice were recorded [43]. Mice were considered protected after 30 min of treatment.

### 4.20. Diuretic Activity

The mice were restricted from food 18 h before the start of the experiment, with access to water. After 45 min of drug administration, 1 mL of saline solution (sodium = 154 mmol/L, chlorine = 154 mmol/L) was orally administered. Each mouse was placed in metabolic cages for 6 h for urine collection [44].

### 4.21. Evaluation of the Possible Mechanism of the Diuretic Activity

A group of mice was administered with 60 mg/kg i.p. L-NAME (a non-selective nitric oxide synthase inhibitor, NOS); another group was administered with 5 mg/kg i.p. indomethacin (prostaglandin synthesis inhibitor). After 15 min, 100 µL of 100 mg/kg p.o. HE was administered. Subsequently, after 45 min, 1 mL/kg of saline solution (sodium = 154 mmol/L, chlorine = 154 mmol/L) was administered orally. Urine was collected in a metabolic cage, and cumulative urine output was measured at 6 h after administration.

### 4.22. Statistical Analysis

The relative frequencies of the ethnomedicinal information were analyzed by the Chi-square test (X^2^) to determine the significant differences in the collected data. The antioxidant activity is expressed in mean and standard error. In this in vitro test, data were analyzed using a one-way Analysis of Variance (ANOVA) with a confidence level of 95% with Tukey’s post hoc test (*p* < 0.05). The results of the pharmacological tests are expressed in mean and standard error and were analyzed using a one-way ANOVA with a confidence level of 95% and with a Dunnett’s post-hoc test (*p* < 0.05) to compare each treatment with the control group. Statistica software version 13 (Statsoft Inc., Tulsa, OK, USA) for statistical analysis and Prism software version 8.0.2 (GraphPad, San Diego, CA, USA) for the elaboration of graphs were used in this study.

## 5. Conclusions

This study describes, for the first time, ethnomedicinal information on the orchid *S. tigrina*. The findings validate the medicinal use of *S. tigrina* as a source of compounds with anxiolytic-like and diuretic effects. HE showed anxiolytic-like activity without producing hypnotic, sedative, and locomotor impairments, which are adverse effects shown by CNZ. The anxiolytic-like effects shown by 100 mg/kg were likely those exerted by 1.5 mg/kg CNZ in three models (light–dark box, elevated plus maze, and hole board). The possible mechanisms of the anxiolytic-like effects of HE are due to the involvement of the gabaergic, adrenergic, and serotonergic systems. HE induced diuretic effects with the excretion of sodium and, to a lesser extent, potassium. The possible mechanism of the diuretic action of HE is through the nitric oxide.

## Figures and Tables

**Table 1:** Chemical composition of the HE obtained by GC-MS analysis.

Peak No.	RT ^1^ (min)	Name of the Compound	Molecular Formula	Molecular Weight (g/mol)	Peak Area (%)	Compound Nature
1	7.980	Acetic acid, [(tert-butyldimethylsilyl)oxy]-, tert-butyldimethylsilyl ester	C_14_H_32_O_3_Si_2_	304.57	1.11	Ester
2	8.621	Methyl-2-iodobenzoate	C_8_H_7_IO_2_	262.04	1.30	Ester
3	8.75	2,3,4-Tris[(trimethylsilyl)oxy]butanal	C_13_H_32_O_4_Si_3_	336.65	2.40	Alkane
4	8.840	(4S,5R)-4-trimethylsilyloxy-5-(trimethylsilyloxymethyl)oxolan-2-one	C_11_H_24_O_4_Si_2_	276.48	0.80	Ester
5	8.920	5-methylsulfanyl-3-phenyl-1,2-oxazole	C_10_ H_9_ NOS	191.25	0.85	Alkaloid
6	9.302	trimethylsilyl 2,3,4-tris(trimethylsilyloxy)butanoate	C_16_H_40_O_5_Si_4_	424.8	0.65	Phenolic acids
7	10.041	Triethyl(1,2,3,4-tetrahydronaphthyl)silane	C_16_H_26_Si	246.47	0.92	Alkene
8	10.062	Neophytadiene	C_20_H_38_	278.5	0.27	Diterpene
9	10.098	2,3,4,5-Tetrahydroxypentanoic acid-1,4-lactone, tris(trimethylsilyl)-	C_14_H_32_O_5_Si_3_	364.66	0.97	lactones, coumarin
10	10.135	2,3-Dimethoxy-1-phenyl-5,5-dimethylcyclopentene	C_16_H_22_O_2_	246.35	1.33	Alkene
11	10.208	methyl 3-(4-trimethylsilyloxyphenyl)propanoate	C_13_H_20_O_3_Si	252.38	1.15	Fatty acid
12	10.270	2-Methyl-5-(2′,4′,6′-trimethylphenyl)tetrahydrofuran	C_14_H_20_O	204.313	1.23	Ether
13	10.315	Trimethylsilyl laurate	C_15_H_32_O_2_Si	272.50	0.12	Ester
14	10.741	(Z)-3-(1-Butylidene)phthalide	C_12_H_12_O_2_	188.22	2.36	Cumarin
15	10.799	n-Heptadecane	C_17_H_36_	240.5	0.00	Alkane
16	10.845	Benzopyrido(2,1-a)isoindole	C_12_H_9_N	167.21	1.73	Alkaloid
17	11.286	3-(methylthio)benzofuro [3,2-e]-1,2,4-triazine	C_10_H_7_N_3_OS	217.25	1.39	Alkaloid
18	11.34	Hydrocinnamic acid, p-(trimethylsiloxy)-, trimethylsilyl ester	C_15_H_26_O_3_Si_2_	310.54	0.46	Phenolic acids
19	11.39	Trimethylsilyl 2,3,4,5-tetrakis-O-(trimethylsilyl)pentonate	C_20_H_50_O_6_Si_5_	527.00	0.63	Trimethylsilyl esters
20	11.439	5-Trichloromethyl-3-[1-(cyanothio)ethyl]-4,5-dihydroisoxazol-5-ol	C7H_7_Cl_3_N_2_O_2_S	288.00	1.20	Alkaloid
21	12.122	Tetradecanoic acid, trimethylsilyl ester	C_17_H_36_O_2_Si	300.6	0.22	Organic acid
22	12.16	1,2,3,4,6-Pentakis-O-(trimethylsilyl)hexopyranose	C_21_H_52_O_6_Si_5_	541.1	2.21	Carbohydrate
23	12.35	D-(-)-Tagatose, pentakis(trimethylsilyl) ether	C_21_H_52_O_6_Si_5_	541.1	0.27	Carbohydrate
24	12.40	β-D-(+)-Mannopyranose, pentakis(trimethylsilyl) ether	C_21_H_52_O_6_Si_5_	541.1	0.53	Carbohydrate
25	12.909	Cinnamic acid, p-(trimethylsiloxy)-, trimethylsilyl ester	C_15_H_24_O_3_Si_2_	308.52	0.73	Phenolic acids
26	12.959	n-Pentanoic acid, trimethylsilyl ester	C_18_H_38_O_2_Si	314.6	0.10	Fatty acid
27	12.981	Gallic acid—tetrakis(trimethylsilyl) derivative	C_31_H_62_O_5_Si_4_	627.2	1.47	Flavonoids
28	13.087	D-Glucose, pentakis-O-(trimethylsilyl)-	C_21_H_52_O_6_Si_5_	541.1	0.51	Carbohydrate
29	13.767	Trimethylsilyl palmitate	C_19_H_40_O_2_Si	328.6	0.64	Fatty acid
30	14.739	(2E)-3,7,11,15-Tetramethyl-2-hexadecenyl trimethylsilyl ether	C_23_H_48_OSi	368.7	1.72	Alkane
31	15.042	Linoleic acid trimethylsilyl ester	C_21_H_40_O_2_Si-	352.6	0.82	Fatty acid
32	15.085	trans-9-Octadecenoic acid, trimethylsilyl ester	C_21_H_42_O_2_Si	354.6	0.86	Fatty acid
33	15.26	Trimethylsilyl stearate	C_21_H_44_O_2_Si	356.7	2.728	Fatty acid
34	16.207	β-benzyl-D-glucopyranoside-tetrakis(trimethylsilyl)-ether	C_25_H_50_O_6_Si_4_	559.0	0.73	Carbohydrate
35	17.51	5,12-Dimethoxy-2,3,8,9-tetramethoxybenzo[c]phenanthridin-6(5H)-one	C_22_H_23_NO_6_	397.427	1.98	Alkaloid
36	17.812	Sucrose, octakis(trimethylsilyl) ether	C_36_H_86_O_11_Si_8_	919.745	2.49	Carbohydrate
37	18.030	3-(2′,2′-Diphenylethenyl)-2,3-dihydro-1H-benzo[e]isoindol-1-one	C_26_H_19_NO	361.444	0.18	Alkaloid
38	19.590	1,4-Diphenyl-2-[N-(methylcarbazol-2′-yl)amino]-2-butene-1,4-dione	C_29_H_23_N_2_O_2_	431.515	0.45	Alkaloid

^1^ RT: retention time in min.

**Table 2:** Chemical composition of the PE obtained by GC-MS analysis.

Peak No.	RT ^1^ (min)	Name of the Compound	Molecular Formula	Molecular Weight (g/mol)	Peak Área (%)	Compound Nature
1	7.18	Melibiose	C_12_H_22_O_11_	342.30	0.21	Carbohydrate
2	8.98	4-Acetylpyrazole	C_5_H_6_N_2_O	110.11	0.90	Alkaloid
3	9.08	(R)-2-(2′-hydroxyethoxy)-2-hydroxymethyl-1,4-dioxane	C_7_H_14_O_3_	146.186	0.19	Eter
4	9.36	Tetracosane	C_24_H_50_	338.7	0.05	Alkane
5	10.73	Per(trimethylsilyl)-D-arabinose	C_17_H_42_O_5_Si_4_	438.9	0.13	Carbohydrate
6	10.84	5-(2-Oxobutyl)-3-phenyl-2-isoxazoline	C_13_H_17_N_1_O_2_	219.284	0.46	Alakaloid
7	11.13	(3SR,4SR)-4-[(RS)-1-Hydroxy-3-bentenyl]-1-(p-methoxyphenyl)-3-(propenyl)-2-azetidinone methanesulfonate	C_17_H_21_NO_6_S	367.71	0.24	Alkene
8	11.57	p-(N,N-Dimethylamino)phenylethynyl]dimesitylborane	C_28_H_32_BN	393.4	0.04	Alkene
9	11.61	Undecanoic acid isopropyl ester, 10-hydroxy-11-morpholin-4-yl-	C_18_H_35_NO_4_	329.5	0.08	Fatty acid
10	11.63	D-(-)-Fructofuranose, pentakis(trimethylsilyl) ether (isomer 1)	C_21_H_52_O_6_Si_5_	541.061	0.12	Carbohydrate
11	11.72	D-(-)-Fructofuranose, pentakis(trimethylsilyl) ether (isomer 2)	C_21_H_52_O_6_Si_5_	541.061	0.20	Carbohydrate
12	11.73	D-(-)-Tagatofuranose, pentakis(trimethylsilyl) ether (isomer 1)	C_21_H_52_O_6_Si_5_	541.061	0.04	Carbohydrate
13	11.78	1-Phenylpyrrolo [2,1,5-cd]indolizine	C_16_H_12_N	218.27	0.08	Alkaloid
14	11.97	1-(p-Acetylbenzoyl)pyrrolidine	C_8_H_10_N_4_O_2_	189.25	0.39	Alkaloid
15	12.09	Caffeine	C_8_H_10_N_4_O_2_	194.19	0.31	Alkaloid
16	12.34	D-Psicose, pentakis(trimethylsilyl) ether	C_22_H_55_NO_6_Si_5_	570.102	0.41	Carbohydrate
17	12.40	β-D-(+)-Mannopyranose, pentakis(trimethylsilyl) ether	C_21_H_52_O_6_Si_5_	541.061	0.37	Carbohydrate
18	12.47	Ethyl 4-(2-Hydroxymethylphenyl)butanoate	C_13_H_18_O_3_	222.277	0.19	Alkane
19	12.87	Per(trimethylsilyl)-D-mannose	C_21_H_52_O_6_Si_5_	541.061	1.22	Carbohydrate
20	13.08	β-D-Glucopyranose, 1,2,3,4,6-pentakis-O-(trimethylsilyl)-	C_21_H_52_O_6_Si_5_	541.061	0.25	Carbohydrate
21	13.09	3,4-Dihydro-2-methylnaphthalene-3,4-diol	C_11_H_10_O_2_	174.20	0.43	Phenolic
22	13.75	Trimethylsilyl palmitate	C_19_H_40_O_2_Si	328.6	0.14	Fatty acid
23	15.032	Linoleic acid trimethylsilyl ester	C_21_H_38_O_2_Si	350.610	0.22	Fatty acid
24	15.25	Trimethylsilyl stearate	C_21_H_44_O_2_Si	356.7	0.13	Fatty acid
25	17.82	Sucrose, octakis(trimethylsilyl) ether	C_36_H_86_O_11_Si_8_	919.745	0.16	Carbohydrate

^1^ RT: retention time in min.

**Table 3:** Effects of HE and PE extracts of *S. tigrina* on OFT.

Treatment	Total Distance (cm)	Resting Time (s)	Time in Central Squares (s)	Distance in Central Squares (cm)
Vehicle	2095.82 ± 157.64	58.04 ± 6.13	19.88 ± 1.57	252.80 ± 26.36
CNZ 1.5 mg/kg	764.89 ± 35.69 *	154.51 ± 4.43 *	48.04 ± 5.64 *	516.28 ± 28.34 *
PE 10 mg/kg	2087.44 ± 90.47	63.35 ± 4.76	17.93 ± 1.48	255.06 ± 32.84
PE 50 mg/kg	2132.38 ± 95.71	62.20 ± 2.20	19.26 ± 0.90	303.17 ± 24.67
PE 100 mg/kg	2120.15 ± 147.06	60.41 ± 3.50	28.24 ± 3.41	369.92 ± 19.09 *
HE 10 mg/kg	1923.22 ± 105.43	60.52 ± 8.90	24.70 ± 3.69	290.22 ± 44.15
HE 50 mg/kg	2212.34 ± 121.45	64.24 ± 7.31	29.20 ± 4.38	351.89 ± 31.01
HE 100 mg/kg	2064.02 ± 109.21	63.24 ± 8.02	37.30 ± 3.11 *	432.22 ± 15.17 *

The data were presented as mean ± SEM; n = 10. The data were examined by one-way analysis of variance (ANOVA) post hoc Dunnett’s test using Statistica software version 13. * *p* < 0.05 compared to the vehicle group. PE: pseudobulb ethanol extract; HE: leaf ethanol extract; CNZ: clonazepam; and Vehicle: saline solution.

**Table 4:** Anticonvulsant activities of PE and HE.

Treatments	Onset of Convulsion (s)	Duration of Convulsion (s)	Mortality (%)
Vehicle	58 ± 3.87	155 ± 15.86	100
CNZ 1.5 mg/kg	0 *	0 *	0
PE 10 mg/kg	63.5 ± 8.09	160.66 ± 18.62	100
PE 50 mg/kg	59.16 ± 6.98	166.83	100
PE 100 mg/kg	72.66 ± 5.38	130.66 ± 19.42	100
HE 10 mg/kg	59 ± 5.64	160.50 ± 24.47	100
HE 50 mg/kg	76.66 ± 15.21	130.50 ± 23.14	100
HE 100 mg/kg	112.83 ± 20.89 *	41 ± 1.74 *	100

The data are presented as mean ± SEM, n = 8. The data were examined by one-way analysis of variance (ANOVA) post hoc Dunnett’s test using Statistica software version 13. * *p* < 0.05 compared to the vehicle group. PE: pseudobulb ethanol extract; HE: leaf ethanol extract; CNZ: clonazepam; and Vehicle: saline solution.

## Data Availability

Data is contained within the article.

## References

[1] World Health Organization (WHO) (2022). World Mental Health Report. Transforming Mental Health for All.

[2] Shahrajabian M.H. (2022). Powerful Stress Relieving Medicinal Plants for Anger, Anxiety, Depression, and Stress During Global Pandemic. Recent Pat. Biotechnol..

[3] Alonso-Castro A.J., Ruiz-Padilla A.J., Ramírez-Morales M.A., Alcocer-García S.G., Ruiz-Noa Y., Ibarra-Reynoso L.D.R., Solorio-Alvarado C.R., Zapata-Morales J.R., Mendoza-Macías C.L., Deveze-Álvarez M.A. (2019). Self-treatment with herbal products for weight-loss among overweight and obese subjects from central Mexico. J. Ethnopharmacol..

[4] Kaur H., Sena S., Jha P., Lekhak M.M., Singh S.K., Goutam U., Arencibia A.D., Kumar V. (2022). *Arundina graminifolia* (D.Don) Hochr. (Orchidaceae): A review of its medicinal importance, phytochemistry and pharmacology activities. S. Afr. J. Bot..

[5] Fonmboh D.J., Fokunang T.E., Ndasi N.P., Ngangmou N.T., Herve B., Tita B.L., Nubia K.C., Awah T.M., Aba E.R., Fokunang C.N. (2021). An Overview of the Ethnobotanic, Ethnopharmacological and Medicinal Importance of Edible Wild Root Tuber Orchids in Cameroon. Asian J. Biotechnol. Bioresour. Technol..

[6] Arya S.S., Rookes J.E., Cahill D.M., Lenka S.K. (2021). Vanillin: A review on the therapeutic prospects of a popular flavouring molecule. Adv. Tradit. Med..

[7] Khatun F., Nasrin N., Monira S., Asaduzzaman M., Apu A.S. (2013). Assessment of neuropharmacological and analgesic potentials of *Geodorum densiflorum* (Lam.) schltr root extracts in experimental animals. Pharmacologyonline.

[8] Watanabe K., Tanaka R., Sakurai H., Iguchi K., Yamada Y., Hsu C.S., Sakuma C., Kikuchi H., Shibayama H., Kawai T. (2007). Structure of cymbidine A, a monomeric peptidoglycan-related compound with hypotensive and diuretic activities, isolated from a higher plant, *Cymbidium goeringii* (Orchidaceae). Chem. Pharm. Bull..

[9] Vergara-Galicia J., Ortiz-Andrade R., Rivera-Leyva J., Castillo-España P., Villalobos-Molina R., Ibarra-Barajas M., Gallardo-Ortiz I., Estrada-Soto S. (2010). Vasorelaxant and antihypertensive effects of methanolic extract from roots of *Laelia anceps* are mediated by calcium-channel antagonism. Fitoterapia.

[10] Dorado-Martinez C. (2020). Ethnopharmacology, Mexico’s therapeutic prolificacy for the sustainable social development. Ecocience Int. J..

[11] Gonzalez-Rivera M.L., Barragan-Galvez J.C., Gasca-Martínez D., Hidalgo-Figueroa S., Isiordia-Espinoza M., Alonso-Castro A.J. (2023). In Vivo Neuropharmacological Effects of Neophytadiene. Molecules.

[12] Gerlach G., Stanhopeinae Mesoamericanae V. (2009). El Aroma Floral de las Stanhopeas de Mexico. Lankesteriana Int. J. Orchid..

[13] Cano-Asseleih L.M., Menchaca-García R.A., Ruiz-Cruz J.Y.S. (2015). Ethnobotany, Pharmacology and Chemistry of Medicinal Orchids from Veracruz. J. Agric. Sci. Technol. A.

[14] Martínez M., García M., Rebeca A. (2007). Efecto de los compuestos orgánicos en la propagación in vitro de *Stanhopea Tigrina* Bateman (Orchidaceae). For. Veracruzana.

[15] Salazar-Cerezo S., Martinez-Montiel N., Cruz-Lopez M.D.C., Martinez-Contreras R.D. (2018). Fungal diversity and community composition of culturable fungi in *Stanhopea trigrina* cast gibberellin producers. Front. Microbiol..

[16] Jiménez-Romero E., Moreno-Vera A., Villacis-Calderon A., Rosado-Sabando J., Morales-Moreira D., Bravo-Bravo A. (2019). Estudio etnobotánico y comercialización de plantas medicinales del bosque protector Murocomba y su área de influencia del cantón Valencia, Ecuador. Cienc. Y Tecnol. Agropecu..

[17] Marasco D., Vicidomini C., Krupa P., Cioffi F., Huy P.D.Q., Li M.S., Florio D., Broersen K., De Pandis M.F., Roviello G.N. (2021). Plant isoquinoline alkaloids as potential neurodrugs: A comparative study of the effects of benzo[c]phenanthridine and berberine-based compounds on β-amyloid aggregation. Chem. Biol. Interact..

[18] Ruwizhi N., Aderibigbe B.A. (2020). Cinnamic acid derivatives and their biological efficacy. Int. J. Mol. Sci..

[19] Wu Z.-L., Wang Q., Wang J.-X., Dong H.-Y., Xu X.-K., Shen Y.-H., Li H.-L., Zhang W.-D. (2018). Vlasoulamine A, a Neuroprotective [3.2.2] Cyclazine Sesquiterpene Lactone Dimer from the Roots of *Vladimiria souliei*. Org. Lett..

[20] Peerapen P., Thongboonkerd V. (2018). Caffeine in Kidney Stone Disease: Risk or Benefit?. Adv. Nutr..

[21] Jee H.J., Lee S.G., Bormate K.J., Jung Y.-S. (2020). Effect of Caffeine Consumption on the Risk for Neurological and Psychiatric Disorders: Sex Differences in Human. Nutrients.

[22] Morales-Sánchez V., Rivero-Cruz I., Laguna-Hernández G., Salazar-Chávez G., Mata R. (2014). Chemical composition, potential toxicity, and quality control procedures of the crude drug of *Cyrtopodium macrobulbon*. J. Ethnopharmacol..

[23] Hsieh W.-T., Tsai H.-Y., Hsieh M.-T., Chen C.-F. (1993). Analgesic and anti-inflammatory effects of *Nervilia purpurea* and its active components. J. Tradit. Chin. Med..

[24] Singh A., Kukreti R., Saso L., Kukreti S. (2019). Oxidative Stress: A Key Modulator in Neurodegenerative Diseases. Molecules.

[25] Bourin M. (2015). Animal models for screening anxiolytic-like drugs: A perspective. Dialogues Clin. Neurosci..

[26] Kagota S., Morikawa K., Ishida H., Chimoto J., Maruyama-Fumoto K., Yamada S., Shinozuka K. (2021). Vasorelaxant effects of benzodiazepines, non-benzodiazepine sedative-hypnotics, and tandospirone on isolated rat arteries. Eur. J. Pharmacol..

[27] Kraeuter A.-K., Guest P.C., Sarnyai Z. (2019). The Elevated Plus Maze Test for Measuring Anxiety-Like Behavior in Rodents. Methods Mol. Biol..

[28] Sarkar D. (2020). A Review of Behavioral Tests to Evaluate Different Types of Anxiety and Anti-anxiety Effects. Clin. Psychopharmacol. Neurosci..

[29] Farzamfard P., Rezayof A., Alijanpour S. (2022). Ventral hippocampal NMDA receptors mediate the effects of nicotine on stress-induced anxiety/exploratory behaviors in rats. Neurosci. Lett..

[30] Kawanabe S., Mori M., Harada H., Murata Y., Ohe K., Enjoji M. (2023). Upregulations of α(1) adrenergic receptors and noradrenaline synthases in the medial prefrontal cortex are associated with emotional and cognitive dysregulation induced by post-weaning social isolation in male rats. Neurosci. Lett..

[31] Wahis J., Holt M.G. (2021). Astrocytes, Noradrenaline, α1-Adrenoreceptors, and Neuromodulation: Evidence and Unanswered Questions. Front. Cell. Neurosci..

[32] Pędzich B.D., Rubens S., Sekssaoui M., Pierre A., Van Schuerbeek A., Marin P., Bockaert J., Valjent E., Bécamel C., De Bundel D. (2022). Effects of a psychedelic 5-HT2A receptor agonist on anxiety-related behavior and fear processing in mice. Neuropsychopharmacology.

[33] Schönfeld L.-M., Dooley D., Jahanshahi A., Temel Y., Hendrix S. (2017). Evaluating rodent motor functions: Which tests to choose?. Neurosci. Biobehav. Rev..

[34] Kumar V., Bhat Z.A., Kumar D. (2013). Animal models of anxiety: A comprehensive review. J. Pharmacol. Toxicol. Methods.

[35] Kehrenberg M.C.A., Bachmann H.S. (2022). Diuretics: A contemporary pharmacological classification?. Naunyn-Schmiedeberg’s Arch. Pharmacol..

[36] Schlickmann F., Boeing T., Mariano L.N.B., da Silva R.C.M.V.A.F., da Silva L.M., de Andrade S.F., de Souza P., Cechinel-Filho V. (2018). Gallic acid, a phenolic compound isolated from *Mimosa bimucronata* (DC.) Kuntze leaves, induces diuresis and saluresis in rats. Naunyn-Schmiedeberg’s Arch. Pharmacol..

[37] Merenzon M.A., Hincapie Arias E., Bhatia S., Shah A.H., Higgins D.M.O., Villaverde M., Belgorosky D., Eijan A.M. (2023). Nitric oxide synthase inhibitors as potential therapeutic agents for gliomas: A systematic review. Nitric Oxide Biol. Chem..

[38] Satoh N., Nakamura M., Suzuki A., Tsukada H., Horita S., Suzuki M., Moriya K., Seki G. (2017). Effects of Nitric Oxide on Renal Proximal Tubular Na+ Transport. BioMed Res. Int..

[39] Munteanu I.G., Apetrei C. (2021). Analytical Methods Used in Determining Antioxidant Activity: A Review. Int. J. Mol. Sci..

[40] Secretaría de Agricultura, Ganadería, Desarrollo Rural, Pesca y Alimentación (2001). Norma Oficial Mexicana NOM-062-ZOO-1999, Especificaciones Técnicas para la Producción, Cuidado y uso de los Animales de Laboratorio.

[41] OECD (2022). Test No. 425: Acute Oral Toxicity: Up-and-Down Procedure, OECD Guidelines for the Testing of Chemicals.

[42] Park C.W., Hong K.B., Suh H.J., Ahn Y. (2023). Sleep-promoting activity of amylase-treated Ashwagandha (*Withania somnifera* L. Dunal) root extract via GABA receptors. J. Food Drug Anal..

[43] de Oliveira C.C., de Oliveira C.V., Grigoletto J., Ribeiro L.R., Funck V.R., Grauncke A.C.B., de Souza T.L., Souto N.S., Furian A.F., Menezes I.R.A. (2016). Anticonvulsant activity of β-caryophyllene against pentylenetetrazol-induced seizures. Epilepsy Behav..

[44] Arana-Argáez V., Alonso-Castro A.J., Yáñez-Barrientos E., Euan-Canto A., Torres-Romero J.C., Isiordia-Espinoza M.A., Brennan-Bourdon L.M., Juárez-Vázquez M.D.C., González-Ibarra A.A. (2021). In vitro and in vivo anti-inflammatory effects of an ethanol extract from the aerial parts of *Eryngium carlinae* F. Delaroche (Apiaceae). J. Ethnopharmacol..

